# Safe Application of 75% Trifloxystrobin–Tebuconazole as Water-Dispersible Granules in Paddy Based on Residue and Dietary Risk Assessment

**DOI:** 10.3390/molecules29010163

**Published:** 2023-12-27

**Authors:** Siwei Wang, Yanping Liu, Manshan Zhu

**Affiliations:** 1 Plant Protection Research Institute, Guangdong Academy of Agricultural Sciences, Key Laboratory of Green Prevention and Control on Fruits and Vegetables in South China Ministry of Agriculture and Rural Affairs, Guangdong Provincial Key Laboratory of High Technology for Plant Protection, Guangzhou 510640, China; wangsiwei@gdaas.cn; 2 Rice Research Institute, Guangdong Academy of Agricultural Sciences/Guangdong Key Laboratory of New Technology in Rice Breeding/Guangdong Rice Engineering Laboratory/Key Laboratory of Genetics and Breeding of High Quality Rice in Southern China (Co-Construction by Ministry and Province), Ministry of Agriculture and Rural Affairs, Guangzhou 510640, China

**Keywords:** trifloxystrobin and tebuconazole, paddy, residue distribution, risk assessment, dissipation dynamics

## Abstract

The present study describes the development of a highly effective approach for determining the residue distribution and dissipation of trifloxystrobin and tebuconazole, and their risk assessment in brown rice, husk, straw, and grain using high-performance liquid chromatography–tandem mass spectrometry (HPLC-MS/MS). The current study provides considerable novel information regarding the safe utilization of a mixture of trifloxystrobin and tebuconazole in paddy production. The samples demonstrated a range of mean recoveries between 72% and 86%, with a 1.1–9.2% relative standard deviation (RSD). The limits of quantification (LOQ) and half-lives (t_1/2_) for brown rice, husk, straw, and grain were, respectively, established to be 0.001–0.01 mg/kg and 4.1–7.7 days. The concentrations of terminal residues in the brown rice, husk, straw, and grain were, respectively, found to be 0.02–0.05, 0.03–0.11, 0.02–0.07, and 0.02–0.05 mg/kg after being treated twice at 168.75 g a.i./ha with 21 and 28 days of pre-harvest intervals (PHIs). Trifloxystrobin and tebuconazole presented a non-negligible chronic risk to human subjects, as evidenced by a risk quotient (RQ) value of less than 1.

## 1. Introduction

Rice is considered one of the most essential food crops throughout the world. China cultivates the majority of rice and produces the highest total yield of food crops. Rice production status is inextricably connected to China’s food security concerns; with an annual yield exceeding 200 million tons and a perennial cultivation area of approximately 30 million hectares, it plays an extremely important role in ensuring national food security [1,2]. Although rice serves as a staple food for over 50% of the global population, it is more prevalent in China, where over 65% of the population relies on it [3]. Weeds, pests, and diseases are significant limiting factors that influence rice yield and quality. Rice crops often experience a yield loss rate of 15% to 30% each year due to diseases, pests, and weeds. Rice embryos and seed coats contain an abundance of physiologically active substances and nutrients, exceeding 64%. Furthermore, rice is enriched with a variety of functional factors that exert physiological effects, including trace nutrients like selenium, zinc, and iron, γ-aminobutyric acid (GABA), inositol, glutathione, vitamin E, dietary fiber, N-dihydroceramide, and other functional physiologically active ingredients [3,4].

Rice quality and ecological safety are seriously compromised due to excessive pesticide application caused by the prevalence of rice diseases and pests. Rice production is primarily affected by sheath blight, rice blast, and bacterial stripe disease [5]. To ensure a stable yield and a substantial harvest of rice, the implementation of pesticides for the prevention and control of diseases, pests, and vegetation is an essential strategy [6,7]. The rice industry can achieve several economic benefits by implementing pesticides for disease and pest control, labor investment savings, and disease prevention. Nevertheless, the use of pesticides in an unscientific, unreasonable, and ineffective manner not only results in increased pesticide resistance and the recurrence of pests and diseases, but also significantly compromises the food safety of rice and increases environmental pollution in the production region, ultimately affecting consumer health [8,9].

**Figure 1:**
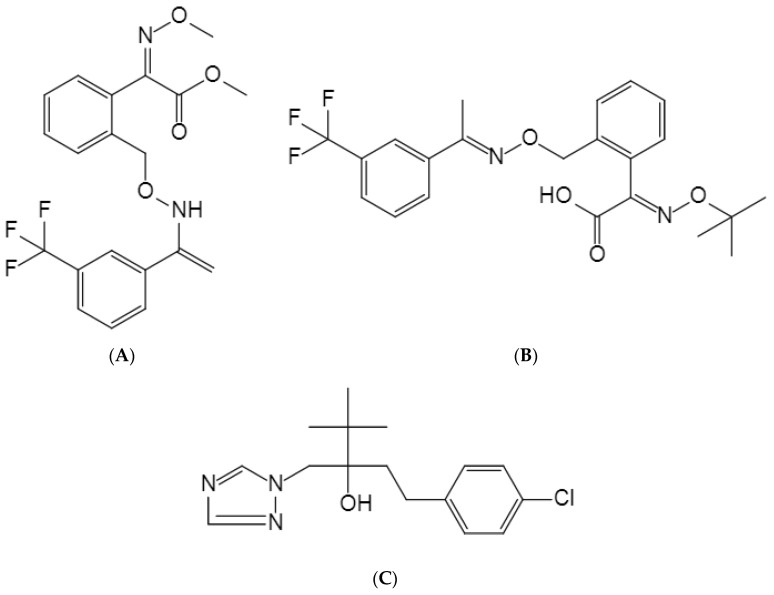
Structure of trifloxystrobin and its metabolite, tebuconazole: (**A**) trifloxystrobin; (**B**) trifloxystrobin acid; (**C**) tebuconazole.

Trifloxystrobin is a respiratory inhibitor from the methoxyacrylic acid class of fungicides. It is resistant to rainwater erosion and is utilized extensively to prevent and control diseases on crops, including apples, cucumbers, potatoes, and tomatoes [10,11]. Its mechanism of action involves blocking the electron transfer chain within mitochondria, thereby inhibiting mitochondrial respiration [12]. Research findings indicate that trifloxystrobin is metabolized by plants into acid products after absorption (CGA321113). Furthermore, the toxicological significance of oximic acid surpasses that of its parent molecule [13]. Trifloxystrobin is a weak allergen and has irritating effects on the eyes [14]. Trifloxystrobin acid is highly toxic to some aquatic non-target organisms [15]. Research has revealed that acidic metabolites are highly soluble in water and might potentially affect ecologically important plant and animal populations [16]. Significant attention must be devoted to subjects pertaining to quality, safety, and environmental concerns. The analysis of pesticide residues in grain crops should therefore be the focus of investigations. Effective pesticide surveillance in rice is crucial for ensuring food safety and minimizing potential health risks for consumers. Tebuconazole, an internally absorbable triazole fungicide with broad-spectrum activity, is an effective agent that was developed in Germany by Bayer AG. It inhibits the growth and development of pathogenic bacteria by interfering with the biosynthesis of pathogenic ergosterol, which consequently affects the structure and function of pathogenic cell membranes. Tebuconazole is predominantly applied via seed treatment or foliar spraying to control a variety of diseases, including rust, root rot, powdery mildew, and smut [17,18]. The development of cross-resistance between trifloxystrobin and tebuconazole is not common because of their different mechanisms of action (The structural formula is shown in Figure 1).

Currently, the primary detection methods for trifloxystrobin and tebuconazole residues include liquid chromatography (HPLC) [19], gas chromatography–mass spectrometry (GC) [20], and liquid chromatography–mass spectrometry (HPLC-MS/MS) [21]. However, this investigation employed HPLC-MS/MS to detect three pesticides, because trifloxystrobin acid is required for this instrument. In recent years, HPLC-MS/MS has emerged as a highly effective method for the identification and quantification of pesticide residues. The aforementioned approach has demonstrated significant benefits in terms of its resolution, selectivity, and analytical capabilities. Consequently, it has become possible to detect an extensive variety of specific pesticides in diverse food matrices. Instrumental analysis and sample pre-treatment are two steps to analyzing pesticide residues. It is necessary to employ suitable cleaning techniques to minimize the potential for the interference effects of matrix and instrument contamination, thus assuring precise quantification. Therefore, it is imperative to establish an efficient approach to the detection of pesticides in rice to mitigate potential health risks and improve international trade. QuEChERS (quick, easy, cheap, effective, rugged, and safe) is characterized by its stability, simplicity, cost-effectiveness, sustainability, and outstanding efficiency [22]. Tebuconazole and trifloxystrobin have been frequently utilized in the cultivation of various crops, which include mango [11], tomato [21], peanut oil [13], ginger [23], and cowpea [23]. Pesticides are usually transformed into different metabolites after application to crops and soils, and these metabolites might have more toxicity and persistence than their parent pesticides. The main metabolite of chlorpyrifos is chlorpyrifos-methyl (TCP), which has high water solubility and migration capacity. It also has stronger toxicity and a longer half-life than chlorpyrifos [24]. However, many reports on residues’ behaviors and dissipation in crops are focused on the parent of trifloxystrobin. It is essential to conduct research on metabolite residue behavior. To date, scientific research pertaining to the concurrent validation of tebuconazole, trifloxystrobin, and their metabolites in the paddy matrix, as well as the evaluation of their residual properties, is limited. Therefore, it is imperative to investigate the residual degradation, distribution, and dietary hazards associated with trifloxystrobin and tebuconazole in paddy.

The primary goal of the current study was to develop a highly efficient and sensitive UPLC-MS/MS method for the detection and quantification of tebuconazole and trifloxystrobin in paddy samples. The study also evaluated the residues and dissipation kinetics of tebuconazole and trifloxystrobin under actual field conditions, encompassing their distribution throughout brown rice, straw, and husk. Finally, the risk presented by tebuconazole and trifloxystrobin in rice was assessed through RQ calculation utilizing the findings of the terminal residue analysis.

## 2. Results and Discussion

### 2.1. Method Verification

The following parameters were utilized to validate the analytical method: linearity, recovery, precision, and accuracy. To evaluate precision and accuracy, the recovery rates were computed. The linearity of samples consisting of husk, brown rice, and vegetation was assessed using matrix-matched standard solutions (0.01–1 mg/L values). Each correlation coefficient (r^2^) was greater than 0.990. Recovery studies were employed to ascertain the accuracy as well as the precision of the experimental measurements. As demonstrated in Table 1, five replicate samples of tebuconazole and trifloxystrobin were analyzed in this investigation at three distinct concentrations.

In the samples of brown rice, straw, and husk, the mean recoveries for trifloxystrobin along with its associated metabolite at all concentrations were observed to be within the acceptable ranges of 77–85% and 72–82%, respectively, with RSDs ranging from 1.8 to 8.7% and 1.1–5.5%. Similarly, the mean recoveries of tebuconazole ranged from 74–86% in the samples, with RSDs of 3.0–9.2%. These findings indicate that the employed methodology corresponds to the established validation standards for the assessment of pesticide residues. The LOQ for trifloxystrobin and its metabolite, tebuconazole, in the brown rice, straw, and husk was observed over a 0.001 to 0.01 mg/kg range. The experimental results indicated that the utilized methodology provided results within the acceptable range, thus demonstrating a high level of reliability and validity.

### 2.2. Dissipation of Trifloxystrobin and Tebuconazole in Paddy

**Figure 2:**
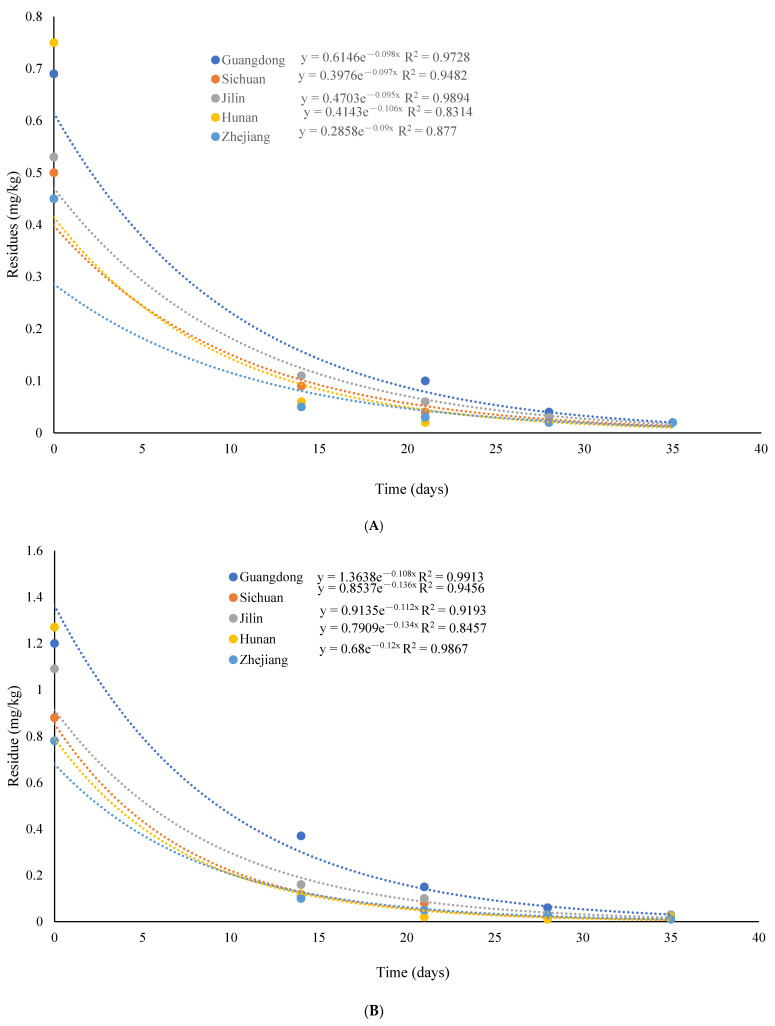
The dissipation pattern of trifloxystrobin and tebuconazole in rice (lateral axis is collected sample time, and longitudinal axis is residues). (**A**) trifloxystrobin; (**B**) tebuconazole.

The patterns of tebuconazole and trifloxystrobin dissipation in rice under open-field conditions are depicted in Figure 2. Trifloxystrobin and tebuconazole were initially present in rice at 0.45–0.75 and 0.78–1.27 mg/kg concentrations, respectively. The regression equation revealed estimates of the half-lives of trifloxystrobin and tebuconazole in rice to be 6.03–7.70 days and 4.07–6.19 days, respectively. After 14 days of application, the concentrations of tebuconazole and trifloxystrobin in rice treated with these substances decreased substantially, reaching 0.10–0.37 and 0.05–0.11 mg/kg, respectively, representing corresponding substantial losses of 79–92% and 69–91%. A significant reduction of 91% was observed in trifloxystrobin and tebuconazole concentrations in rice 35 days after application, resulting in concentrations below 0.03 mg/kg. The five experimental sites displayed divergent climatic conditions, with particular emphasis on tebuconazole. Multiple factors, such as temperature, humidity, pH, and sunlight, can be attributed to the dissipation of residues. These results showed that harvesting can be done safely, especially after 28 days of using the recommended dose of trifloxystrobin and tebuconazole.

### 2.3. Terminal Residues of Trifloxystrobin and Tebuconazole in Paddy

**Figure 3:**
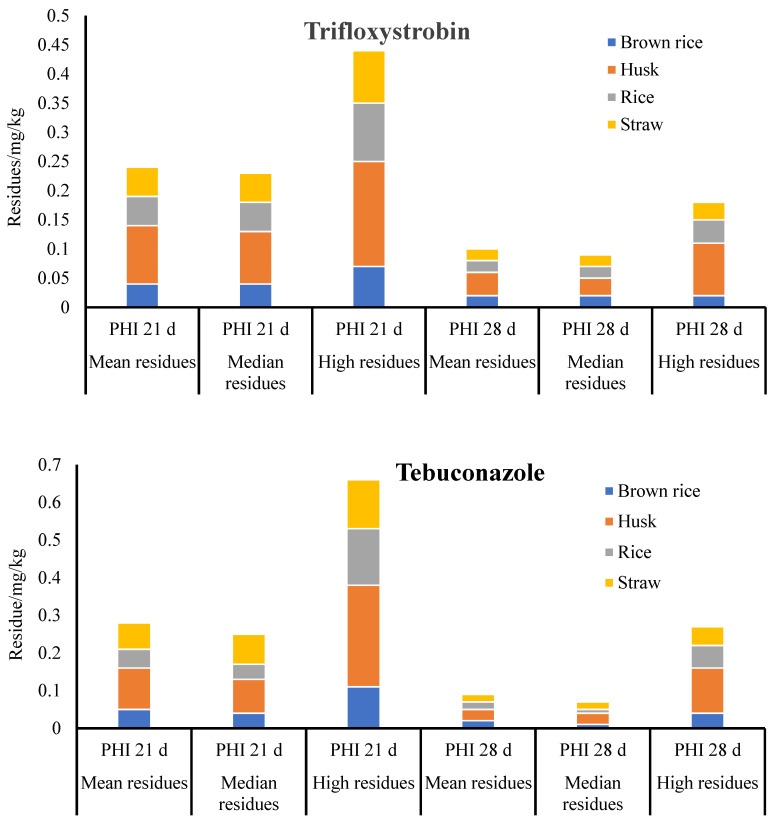
Terminal residues of trifloxystrobin and tebuconazole in brown rice, husk, and rice.

The final residue concentrations of trifloxystrobin and tebuconazole identified in the paddy samples after collection from the treated plots are presented in Figure 3. At the recommended dose of 168.75 g a.i./ha, the terminal residue contents of trifloxystrobin and tebuconazole in brown rice, husk, rice, and straw were determined 21 and 28 days following final application.

The observed levels of trifloxystrobin residue were found to be in the ranges of 0.02 to 0.07 mg/kg, 0.03 to 0.18 mg/kg, 0.02 to 0.10 mg/kg, and 0.02 to 0.09 mg/kg in the brown rice, husk, rice, and straw, respectively, considering PHIs of 21 days. However, the trifloxystrobin residues were significantly decreased in the brown rice (01 to 0.03 mg/kg), husk (00.02 to 0.09 mg/kg), rice (0.01 to 0.04 mg/kg), and straw (0.01 to 0.03 mg/kg) at PHIs of 28 days. Similar trends can be observed in the final residues of tebuconazole and trifloxystrobin. When the PHI was 21 (28) days, the tebuconazole residues in the brown rice, husk, rice, and straw ranged from 0.01 to 0.11 mg/kg (0.01 to 0.04 mg/kg), 0.04 to 0.27 mg/kg (0.01 to 0.12 mg/kg), 0.02 to 0.15 mg/kg (0.01 to 0.06 mg/kg), and 0.02 to 0.13 mg/kg (0.01 to 0.05 mg/kg). The residues of trifloxystrobin acid were <0.01–0.01 mg/kg, 0.01–0.03 mg/kg, <0.01–0.01 mg/kg, and <0.01–0.02 mg/kg in the brown rice, husk, rice, and straw, respectively. The observed concentration trend under various PHIs showed that residues reduced with increasing PHIs.

The husk residues were significantly higher as compared to the brown rice, straw, and rice. The husk is believed to contain a higher quantity of most residues. No pesticide was directly exposed to the rice when a mixed agent consisting of 75% tebuconazole and trifloxystrobin was applied to the husk. Alternatively, according to the *e-Pesticide Handbook* (Version 3.0), the fat-soluble fungicides trifloxystrobin and tebuconazole have log Dow values of 3.7 and 4.5, respectively. Therefore, it is anticipated that the residue will demonstrate an enhanced capacity for absorption. Consequently, it was observed that the contaminant levels that permeated the rice were significantly reduced. Based on the information that is currently available, it appears that the distribution pattern of residual pesticides in husks is significantly higher than in rice [25]. Brown rice, the edible rice part, presents a comparatively minor hazard to human health. However, additional research is required regarding the residual risk when it is used for other purposes. The residues of mepiquat and paclobutrazol are most prevalent in straw, and then husks, and the residues in brown rice are negligible [26]. Numerous variables influence the pesticide distribution in crops, including pesticide properties, environmental conditions, crop characteristics, application methods, and agronomic practices [27].

The median residue values for tebuconazole and trifloxystrobin, as determined by the analysis, were 0.02 and 0.01 mg/kg, respectively, while 0.03 and 0.04 mg/kg were recorded as the maximum residues. Trifloxystrobin had a maximum residual limit of 0.5 mg/kg and tebuconazole of 0.1 mg/kg in rice (2 mg/kg and 0.05 mg/kg in Japan, 5 mg/kg and 1.5 mg/kg in the European Union, and 3.5 mg/kg and none in the USA, respectively). The use of 75% tebuconazole and trifloxystrobin WDGs is suggested for the prevention and management of diseases in rice. The maximum authorized dosage is 168.75 g a.i./ha, with two applications and 28 days of PHI.

### 2.4. Risk Assessment

The RQ method was employed to examine the risk of trifloxystrobin and tebuconazole in paddy in relation to the typical food intake of the Chinese population (Table 2) [28]. The risk assessment of tebuconazole and trifloxystrobin residues in samples of brown rice utilized ADI values obtained from the JMPR report. At the 28-day pre-harvest interval, the STMRs for trifloxystrobin and tebuconazole were, respectively, determined to be 0.02 and 0.01 mg/kg. Chinese consumers were assessed to determine the potential risk associated with prolonged consumption of tebuconazole and trifloxystrobin, accounting for risk percentages of 11.9% and 89.4%. Importantly, these values were less than 100%, showing that trifloxystrobin and tebuconazole applied to paddy did not appear to represent any significant chronic dietary risk to Chinese consumers.

## 3. Material and Methods

### 3.1. Chemicals and Reagents

Trifloxystrobin (99.5%) and tebuconazole (99.3%) standard were purchased from Dr. Ehrenstorfer GmbH (Augsburg, Germany), and trifloxystrobin acid (98.0%) was obtained from FUJIFILM Wako Pure Chemical Corporation (Richmond, VA, USA). Hunan Agricultural University Haite Agrochemical Co., Ltd. (Changsha, China) provided a mixed agent consisting of 75% tebuconazole and trifloxystrobin as water-dispersible granules (WDGs). Thermo Fisher Scientific Inc. (Waltham, MA, USA) supplied methanol, acetonitrile, and HPLC-grade solvents, while chromatographically pure acetonitrile (MeCN) was supplied by Fisher (Clarion County, PA, USA). Sodium chloride (NaCl) and anhydrous magnesium sulfate (MgSO_4_) of analytical grade were provided by Sinopharm Chemical Reagents Co., Ltd. (Shanghai, China). Octadecylsilane (C_18_, 40 μm) sorbent was purchased from Agela Technologies Inc. (Tianjin, China). HPLC-grade formic acid was provided by the Aladdin Industrial Corporation in Shanghai, China. Water was purified utilizing Millipore’s Simplicity UV water-purification system (Bedford, MA, USA).

Stock solutions with a concentration of 1000 mg L^−1^ were prepared for each standard pesticide in acetonitrile and stored at −20 °C. Following this, fresh working standards were prepared for calibration and fortification via dilution of the stock solution. The solvent calibration solutions were prepared via mixing in acetonitrile. The matrix-matched solutions were prepared by combining blank extracts from each matrix with the stocks. All the prepared solutions were stored at 4 °C before use.

### 3.2. Field Trials and Sample Preparation

Dynamic and terminal residues were both analyzed in field examinations which were carried out at 12 different sites, including Guangdong Province (Guangzhou City, China), Sichuan Province (Chengdu City, China), Jilin Province (Changchun City, China), Hunan Province (Hengyang City, China), Zhejiang Province (Shaoxing City, China), Shandong Province (Jinan City, China), Hainan Province (Danzhou City, China), Jiangxi Province (Gaoan City, China), Hubei Province (Wuhan City, China), Anhui Province (Wuhu City, China), Inner Mongolia Autonomous Region (Tongliao City, China), and Guangxi Zhuang Autonomous Region (Nanning City, China) in 2018. In conducting this research, adherence to the “Guideline on Pesticide Residue Trials” (NY/T 788-2004) issued by the Ministry of Agriculture of the People’s Republic of China was strictly followed. The experimental design and implementation of the study were as described on the labels of the pesticide.

The experiment on terminal residue was carried out under the supervision of a field trial supervisor. The suggested dosage of 168.75 g a.i./hectare was applied twice, separated by a period of 7 days between each application. Each experimental plot consisted of a 100 m^2^ area. The experimental design comprised three replicated plots and one control. Collection of respective samples (1 kg plants, or 2 kg ears of paddy) was carried out at preharvest intervals (PHIs) of 21 and 28 days. Sample collection was conducted at multiple locations within each allocation after the last spraying operation. The samples utilized in the analysis of terminal residues consisted of plants, brown rice, and husk. The samples for subsequent analysis were collected utilizing the quartering procedure.

The dissipation of tebuconazole and trifloxystrobin was examined in the paddy by preparing 75% WDGs by dissolving tebuconazole and trifloxystrobin in water. WDGs were applied to the paddy plot at 168.75 g a.i./hectare. Without concurrently administering tebuconazole and trifloxystrobin, a comparative analysis was performed on a plot with similar dimensions. From each plot, plant and ear samples of paddy were collected at 2 h, 14 days, and 35 days following application to examine dissipation. All specimens were stored for subsequent examination at a temperature of −20 °C.

The field plant sample was cut into segments of less than 1 cm in length, thoroughly mixed, divided into two portions of 100 g using the quartering method, placed in sealed plastic bags, appropriately labeled, and stored at −20 °C.

Following the threshing of field rice ear samples, brown rice and rice husk samples need to be roughened with a manual or mechanical threshing device (wet samples should be dried before threshing). Two portions of 100 g of brown rice and two portions of sufficient rice husks were taken after the brown rice and rice husks had been combined separately. They were placed in sealed plastic bags with appropriate labels, and stored at −20 °C for further investigation.

### 3.3. Extraction and Cleanup Procedures

Brown rice samples: To a centrifuge tube (50 mL), we added 10 g of the samples that were ground with a traditional Chinese medicine pestle. Following this, 10 mL of water and 30 mL of acetonitrile containing 0.01% formic acid were transferred to the tube and the contents were subjected to 30 min of vigorous shaking for extraction. Following filtration, 5 g of NaCl was incorporated, and the samples were centrifuged for 5 min at 5000 rpm. For purification purposes, the supernatant (3 mL) was transferred to a 10 mL test tube and subjected to drying in a water bath at 40 °C with nitrogen blowing, and subsequently diluted with 2 mL of methanol. A total of 3 mL methanol-fixed solution was added to a tube (5 mL) containing C18 (50 mg) and anhydrous MgSO_4_ (150 mg) to accelerate the cleanup process. The samples were then centrifuged for 5 min at 5000 rpm after vigorous one-minute agitation. The resultant supernatants were subsequently transferred to an autosampler vial for HPLC-MS/MS analysis after being filtered through a 0.22 μm nylon syringe filter.

Husk and plant samples: To a centrifuge tube (50 mL), we added 5 g of the samples that were ground with a traditional Chinese medicine pestle. Following this, to the tube, we added water (10 mL) and 30 mL of acetonitrile containing 0.01% formic acid, and the contents were subjected to 30 min of vigorous shaking for extraction. Following filtration, 5 g of NaCl was added followed by centrifugation at 5000 rpm for 5 min. For purification, the supernatant (6 mL) was transferred to a 10 mL test tube and allowed to dry in a water bath at 40 °C with nitrogen blowing, followed by dilution with methanol (2 mL). To facilitate the cleanup process, 3 mL of the methanol-fixed solution was transferred into a tube (5 mL) containing anhydrous MgSO_4_ (150 mg) and 50 mg of C18. The materials were then centrifuged for 5 min at 5000 rpm after vigorous shaking for 1 min. The obtained supernatants were subsequently passed through a nylon syringe filter (0.22 μm) and placed in an autosampler vial to be analyzed later through HPLC-MS/MS.

### 3.4. Instrumentation and Analytical Conditions of UPLC

Tebuconazole and trifloxystrobin were extracted through an Agilent LC-1200 HPLC system equipped with an Agilent XDB C18 column (100 × 4.6 mm, with 1.8 μm thickness stationary film) while maintaining a 40 °C temperature in the column oven. A (aqueous formic acid, 0.1%) and B (MeCN) (*v*/*v*, 25/75) were employed in the separation procedure as mobile phases. The flow rate was consistently controlled at 0.25 mL/min with a 1 μL injection volume of the sample.

The samples were analyzed using positive ion mode electrospray ionization (ESI+) on a triple quadrupole Agilent LC1120-MS6400 mass spectrometer (Agilent; Santa Clara, CA, USA). The following instrument parameters were selected: 350 °C for the heating block and 4000 V for the capillary voltage. For the evaluation of tebuconazole and trifloxystrobin via multiple reaction monitoring, nitrogen was employed as the nebulizer as well as collision gas. Table 3 contains a list of the relevant mass spectrometry parameters.

### 3.5. Methodological Validation

The recovery rate (RR), linear equations, and limits of quantification (LOQ) were used to evaluate the reliability and accuracy of the developed methods. Linearity was observed in the developed standards within a concentration range of 0.001 to 0.5 mg L^−1^ concentration. At 0.001, 0.01, or 0.1 mg kg^−1^ concentrations, tebuconazole and trifloxystrobin were artificially introduced into blank food samples to determine the RR values. Five replicates of each concentration were performed to ensure statistical reliability. RSDs were used to assess the methodological precision of this study. The LOQ for each compound (SANTE/11813/2017) was computed utilizing the minimum detected elevated level in the specified matrix.

### 3.6. Statistical Analysis

The kinetics of tebuconazole and trifloxystrobin dissipation in paddy were examined by plotting the concentration of residue vs. time. The best-fit curves of equations were determined by utilizing the correlation coefficient’s maximum squares. Further validation of first-order kinetics was carried out through graphical examination by employing the equation C_t_ = C_0_e^−kt^, where k is the dissipation degradation rate constant, C_t_ denotes the relationship between the quantities of pesticide residue at time t, and C_0_ reflects the initial concentration following application. It was determined that the dissipation half-life (t_1/2_) was equal to ln2/k. All the observed data are presented as means ± standard deviation (SD) after 5 replications.

The RQ approach was used to safely apply tebuconazole and trifloxystrobin by determining the risk and dietary exposure. An RQ value greater than 1 signifies an inadequate pesticide risk, whereas a value less than 1 indicates a negligible risk to human health. Chronic dietary exposure assessment [29] examined the potential risks associated with prolonged consumption. This assessment entailed the computation of exposure levels through the utilization of acceptable daily intake (*ADI*, mg/kg/d) and the median concentrations of pesticide residues on paddy. The formula utilized to calculate the chronic risk quotient (*ADI*%) can be represented as follows:

*ADI*% = (*STMR* × FI)/(*ADI* × *bw*) × 100%,

where FI (kg/day) is the dietary reference intake denoting a specific food type utilized to assess the nutrient consumption patterns of the healthy population in China, *bw* (kg) denotes mean body weight, and *STMR* (mg/kg) is the median residues of supervised trials. The observed *bw* among Chinese adults was determined to be 63 kg, as reported by the China Health and Nutrition Survey.

## 4. Conclusions

A combination of the QuEChERS and HPLC-MS/MS techniques was utilized to determine the residues of tebuconazole and trifloxystrobin in brown rice, rice, and straw. To ascertain the safe application of trifloxystrobin and tebuconazole, an examination was conducted on their dissipation, terminal residues, distribution, and associated risk factors. The results indicate that both trifloxystrobin and tebuconazole dissipated rapidly in rice, with an estimated half-life ranging from 4.1 to 7.7 days. Factors including the water partition coefficient of octanol and aqueous solubility may contribute to the fact that trifloxystrobin and tebuconazole adhere primarily to wax-rich husks after 28 days of application. The final residue concentrations of trifloxystrobin and tebuconazole in brown rice were, respectively, found to be 0.02–0.07 mg/kg and 0.02–0.11 mg/kg, which was lower than the 0.1 mg/kg and 0.5 mg/kg designated as the maximum residue limit in China. The chronic risk quotient score over 1 indicates that the potential hazards to human health posed by trifloxystrobin and tebuconazole at the recommended dosage are non-negligible. The presence of residual quantities of two pesticides, measuring 0.09 and 0.13 mg/kg, in straw necessitates careful evaluation of their safety in the context of animal feed production.

## Figures and Tables

**Table 1:** Performance characteristics of the method for trifloxystrobin and its metabolite, tebuconazole, in the brown rice, husk, and plant.

Pesticide	Matrix	Fortified Level ^a^ (mg/kg)	Average Recovery ^b^ (%, n = 5)	RSD ^c^ (%)	Correlation Coefficient	LOQ (mg/kg)
trifloxystrobin	brown rice	0.01	77	4.3	0.9991	0.001
		0.1	80	3.2		
		1	85	6.3		
	husk	0.01	80	8.7	0.9905	0.005
		0.1	77	2.7		
		1	83	2.3		
	straw	0.01	83	2.9	0.9922	0.005
		0.1	80	5.0		
		1	84	1.8		
trifloxystrobin acid	brown rice	0.01	75	1.6	0.9990	0.005
		0.1	77	1.1		
		1	82	1.6		
	husk	0.01	78	5.5	0.9914	0.01
		0.1	78	1.5		
		1	80	5.0		
	straw	0.01	77	1.4	0.9904	0.01
		0.1	72	2.1		
		1	81	2.0		
tebuconazole	brown rice	0.01	84	3.6	0.9995	0.005
		0.1	86	3.5		
		1	84	3.9		
	husk	0.01	83	9.2	0.9920	0.01
		0.1	78	9.2		
		1	79	8.6		
	straw	0.01	77	3.0	0.9901	0.01
		0.1	74	4.4		
		1	78	4.8		

^a^ The standard fungicide was spiked before the sample grinding. ^b^ The recovery was calculated using the formula Recovery = *C_d_/C_s_* × 100%, where *C_d_* represents the detected concentration and *C_s_* represents the spiked concentration. Results are expressed as mean ± standard deviation (SD) with 95% confidence intervals. ^c^ Mean value of five determinations.

**Table 2:** The risk evaluation of trifloxystrobin and tebuconazole in rice trifloxystrobin tebuconazole.

Pesticide	Food Classification	Fi (kg)	Reference Residue Limits (mg/kg)	Sources	NEDI (mg)	ADI (mg/kg)	Risk Quotient (%)
Trifloxystrobin	Rice and its products	0.2399	0.02	STMR	0.0048	0.04 × 63	
	Flour and its products	0.1385	0.2	China	0.0277		
	Other cereals	0.0233	0.02	China	0.0005		
	Tubers	0.0495	0.2	China	0.0099		
	Dried beans and their products	0.016					
	Dark vegetables	0.0915	0.7	China	0.0641		
	Light vegetables	0.1837	0.3	China	0.0551		
	Pickles	0.0103					
	Fruits	0.0457	3	China	0.1371		
	Nut	0.0039					
	Livestock and poultry	0.0795					
	Milk and its products	0.0263					
	Egg and its products	0.0236					
	Fish and shrimp	0.0301					
	Vegetable oil	0.0327					
	Animal oil	0.0087					
	Sugar and starch	0.0044					
	Salt	0.012	0.05	EU	0.0006		
	Soy sauce	0.009					
	Total	1.0286			0.2997	2.52	11.9
Tebuconazole	Rice and its products	0.2399	0.01	STMR	0.0024	0.03 × 63	
	Flour and its products	0.1385	0.05	China	0.0069		
	Other cereals	0.0233	0.05	China	0.0012		
	Tubers	0.0495	0.02	EU	0.0010		
	Dried beans and their products	0.016					
	Dark vegetables	0.0915	2	China	0.1830		
	Light vegetables	0.1837	7	China	1.2859		
	Pickles	0.0103					
	Fruits	0.0457	3	China	0.1371		
	Nut	0.0039					
	Livestock and poultry	0.0795					
	Milk and its products	0.0263					
	Egg and its products	0.0236					
	Fish and shrimp	0.0301					
	Vegetable oil	0.0327	2	China	0.0654		
	Animal oil	0.0087					
	Sugar and starch	0.0044	0.7	America	0.0031		
	Salt	0.012					
	Soy sauce	0.009	0.7	Japan	0.0063		
	Total	1.0286			1.6899	1.89	89.4

**Table 3:** UPLC-MS/MS parameters for detection of trifloxystrobin and its metabolite, tebuconazole.

Analyte	Precursor Ion (*m*/*z*)	Quantitative Transition (*m*/*z*)	Collision Energy (V)	Qualitative Transition (*m*/*z*)	Collision Energy (V)	Polarity
trifloxystrobin	409.2	186.1	10	206.1	20	positive
trifloxystrobin acid	395.1	186.1	10	148	5	positive
tebuconazole	308.1	70.1	20	125	30	positive

## Data Availability

Data are contained within the article.
